# Healthcare Practitioners' Perspective of Prevailing Awareness on Diabetes Complications: A Questionnaire-Based Pan-India Study

**DOI:** 10.7759/cureus.42568

**Published:** 2023-07-27

**Authors:** Nikhil Tandon, Piya Ballani Thakkar, Jubbin Jacob, Pramila Kalra, Nanditha Arun, Alok Kanungo, Ashish Birla, Ashish Prasad, Mayuri Talathi

**Affiliations:** 1 Endocrinology and Metabolism, All India Institute of Medical Sciences, New Delhi, IND; 2 Diabetes and Endocrinology, Bombay Hospital and Medical Research Centre, Mumbai, IND; 3 Endocrinology, Christian Medical College, Ludhiana, IND; 4 Endocrinology, MS Ramaiah Medical College & Hospital, Bengaluru, IND; 5 Diabetes and Endocrinology, Dr. A. Ramachandran’s Diabetes Hospital, Chennai, IND; 6 Diabetes and Endocrinology, Dr. Kanungo’s Diabetes Centre, Bhubaneswar, IND; 7 Scientific Services, USV Private Limited, Mumbai, IND

**Keywords:** management, prediabetes, asymptomatic diabetes, awareness, diabetes mellitus

## Abstract

The increasing prevalence rate of diabetes mellitus (DM) and the associated long-term complications warrant a need to improve awareness of DM-related complications in the Indian population. Our questionnaire-based pan-India study (April 2021-March 2022) aims to capture the observations of healthcare practitioners (HCPs) on the prevailing level of knowledge and awareness regarding diabetes among their patients. We refer to this as the 90:90:90 program. It aims to achieve 90% awareness, 90% screening and detection of diabetes and prediabetes, and 90% achievement of effective treatment and control. A structured questionnaire was circulated to 1800 HCPs using Google Forms (Google, Mountain View, CA) and Zoom poll questions (Zoom Video Communications, Inc., San Jose, CA) during 125 symposiums. About half (48.6%) of the HCPs observe that less than 40% of their patients are aware of the risk factors of diabetes, and less than 60% of the patients were aware of its cardiovascular complications. About 92-95% of the HCPs recommend screening for diabetes in adults over 30 years of age and suggest the inclusion of a blood glucose estimate as a fifth vital to be tested during doctor visits. Less than 40% of patients fail to achieve the treatment goal, possibly due to lack of adherence, access to medicines, and financial constraints. Therefore, spreading awareness of DM complications and early screening for DM among adults (>30 years) could help achieve better management and treatment outcomes.

## Introduction

Diabetes mellitus (DM) is a chronic metabolic disease associated with potential complications, including cardiovascular complications, atherosclerosis, and neuropathy [1]. Long-standing DM may result in organ damage and mortality. Early detection and appropriate treatment of DM can help mitigate the associated risks. More than half of the patients with diabetes remain asymptomatic, leading to under-diagnosis of the condition among the population [2]. The American Association of Clinical Endocrinology and the U.S. Preventive Services Task Force (USPSTF) recommend screening for pre-diabetes and diabetes in individuals above the age of 45 years and 35 years, respectively.

In India, socioeconomic diversity contributes to disparities in the diagnosis and healthcare delivery of chronic illnesses, including DM [3]. In the current scenario of the healthcare system in India, the importance of insurance coverage and routine annual health check is still evolving [4]. This can contribute to an increased number of newly diagnosed diabetes and pandemic-induced exacerbation of pre-existing diabetes among the affected individuals [5]. Understanding the level of knowledge among patients with DM and healthcare practitioners (HCPs) can help in adopting improved strategies to prevent long-term complications of DM. Our questionnaire-based study aims to capture the observations of HCPs on the prevailing level of knowledge and awareness regarding diabetes, its risk factors, and complications among patients visiting their respective clinics.

## Materials and methods

We conducted an online survey program in 125 symposiums for a period of one year from April 2021 to March 2022 to document the clinical experience of HCPs in India.

The survey was conducted using two modalities: online Google Forms (Google, Mountain View, CA) and a poll conducted over Zoom (Zoom Video Communications, Inc., San Jose, CA) during regional meetings. A pilot study was conducted by circulating the online questionnaire to HCPs selected at random from different parts of India. The responses were documented and used as a reference to validate the responses during the actual conduct of the survey at different regional meetings. At the end of each regional meeting, the documented responses were projected on the screen along with the pre-validated all-India survey for comparison.

The 90:90:90 program

The theme of the symposiums was to achieve a 90% goal of awareness and screening/detection, 90% of the diagnosed undergoing treatment, and 90% of the population with diabetes control. The survey comprised questions pertaining to awareness (90), screening and detection (90), and treatment and control (90) of diabetes (Table 1).

**Table 1:** The 90:90:90 program – survey questions. * Based on HCPs' perception of sustained glycemic control defined by two of the three criteria: fasting blood sugar < 100, postprandial blood sugar < 160, and glycosylated hemoglobin < 7.0% with minimal to no events of hypoglycemia. HCPs: healthcare practitioners; CVD: cardiovascular disease; T2DM: type 2 diabetes mellitus; LDL-C: low-density lipoprotein cholesterol.

S. No.	Q. No.	Question	Scope
1	1A	What is the current level of awareness regarding risk factors of diabetes among patients and caregivers with whom you interact in your routine practice?	1^st^ 90
2	1B	Where do you think India is lacking compared to its Western counterparts in creating awareness?	1^st^ 90
3	1C	Is there any difference in awareness between the rural and urban populations?	1^st^ 90
4	2A	What could we do to improve the awareness of diabetes in the general Indian population?	1^st^ 90
5	2B	In your practice, who plays an important role in improving awareness amongst your patients? (You may choose multiple options)	1^st^ 90
6	3A	Should diabetes screening be made mandatory in the population over 30 years of age?	1^st^ 90
7	3B	Do you agree that blood glucose level needs to be considered as a 5th vital for all patients visiting clinics or hospitals?	1^st^ 90
8	3C	Do you screen for diabetes when a patient visits for their first cardiology consultation?	1^st^ 90
9	4A	How many % of your patients with heart disease also have diabetes?	1^st^ 90
10	4B	How many % of your patients are aware that CVD is the leading cause of death in T2DM?	1^st^ 90
11	5A	What are the major reasons for late diagnosis/undertreatment of diabetes in India?	2^nd^ 90
12	5B	What % of your patients achieve their treatment goal*?	2^nd^ 90
13	5C	In your clinical practice, what is the most challenging part of achieving treatment goals (i.e., blood glucose, blood pressure, and lipids) in your T2DM patients?	2^nd^ 90
14	6A	What is/are the most common reason(s) for lack of adherence to medication among T2DM patients? (You may choose more than 1 option)	3^rd^ 90
15	6B	According to your clinical experience, what do you think are the top three potential barriers/hindrances to achieving optimal diabetes control among your patients with T2DM?	3^rd^ 90
16	6C	Do you think availability and accessibility to medication play a vital role in treatment adherence?	3^rd^ 90
17	7	In real life, why are patients with diabetes not able to achieve their LDL-C treatment goals? (You may choose multiple options)	3^rd^ 90
18	8	In which areas do you think it is important to provide better educational tools to achieve guideline goals? (You may choose multiple options)	3^rd^ 90

Data collation and analysis

The responses were captured or exported to Microsoft Excel (Microsoft Corp., Redmond, WA), consolidated, and analyzed using Microsoft Excel. All the questions in the survey did not demand mandatory responses. Not receiving a response has not been interpreted or considered for the analysis. The calculations were performed based on the number of responses received for each question instead of the total number of participants. Accordingly, the proportion of responses received is represented as percentages.

## Results

In our survey, a total of 1800 HCPs (north = 20%, east = 4%, west = 16%, and south = 60%) from different regions participated.

Response to awareness-based questions

**Figure 1:**
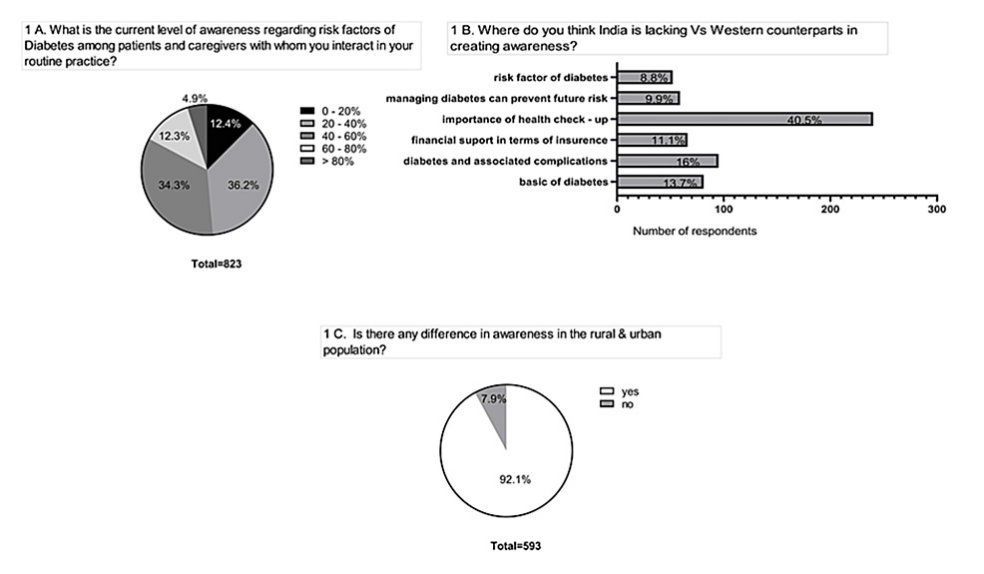
Level of awareness regarding risk factors of diabetes. (A) Clinical setting. (B) India in comparison to Western countries. (C) Urban and rural settings.

We captured the extent of awareness of risk factors among patients with diabetes as observed by practitioners. Almost half (48.6%) of the HCPs responded that only less than 40% of their patients demonstrate an adequate extent of awareness regarding the associated risk factors of diabetes (Figure 1A). HCPs observe a difference in the extent of awareness between the urban and rural populations (Figure 1C). Almost 40% of the HCPs acknowledge that diabetic patients in India do not realize the importance of regular check-ups, and more than 10% of the patient population remains unaware of diabetes-mediated complications (Figure 1B).

**Figure 2:**
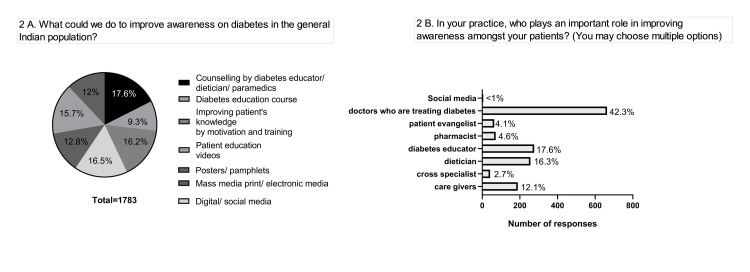
Improving the level of awareness among diabetic patients. (A) General Indian population. (B) Professionals involved in spreading awareness.

All the HCPs propose the need for the spread of awareness of diabetes-mediated complications among the general population. It can be channeled by any mode, including direct counseling or training programs, social media or digital media-based programs, and posters (Figure 2A). About 40% of respondents suggest that doctors play the most important role in providing and enhancing awareness among patients (Figure 2B).

**Figure 3:**
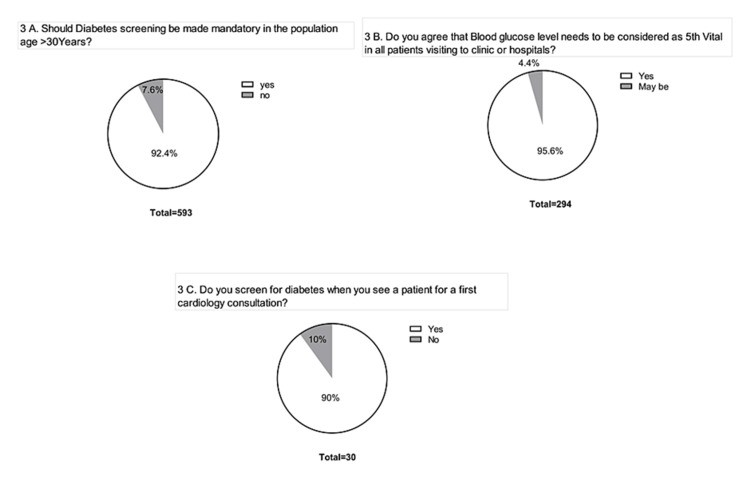
Screening for diabetes. (A) Mandatory screening of diabetes. (B) Blood glucose level as a 5th vital parameter. (C) Diabetes screening for cardiology consultation.

Except for a few, all (93-96%) of the respondents recommend diabetes screening and propose mandatory blood glucose level monitoring for individuals over 30 years of age (Figure 3A). Aligning with this, almost all the HCPs (95%) highlight the need for considering blood glucose level monitoring as the fifth vital for patients visiting the hospitals (Figure 3B). A large proportion of HCPs (90%) screen patients for diabetes when they come for their first cardiology consultation (Figure 3C).

**Figure 4:**
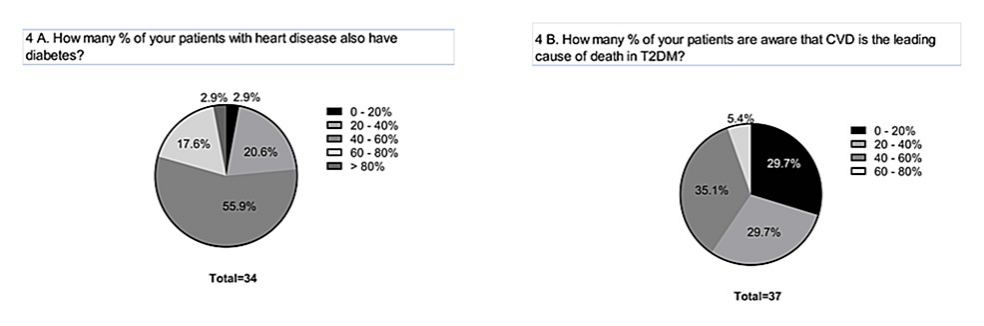
Comorbid condition of diabetes with heart disease. (A) Distribution. (B) Awareness. CVD: cardiovascular disease; T2DM: type 2 diabetes mellitus.

HCPs confirmed that about half of their patients with heart disease also have diabetes, and about 95% of the HCPs responded that less than 60% of their patients are aware of cardiovascular complications as a leading cause of death among type 2 diabetes mellitus (T2DM) patients (Figure 4).

Response to screening and detection-based questions

**Figure 5:**
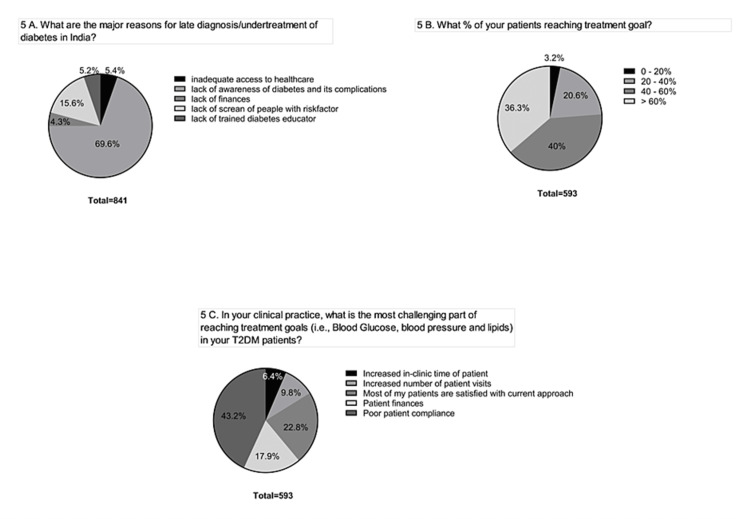
Screening and treatment. (A) Reasons for undertreatment. (B) Patients reaching treatment goals. (C) The challenging part of treatment goals. T2DM: type 2 diabetes mellitus.

Three-fourths of the respondents claim that the major reason for the under-diagnosis of diabetes could be due to a lack of awareness or financial constraints among the patients with T2DM (Figure 5A). Around 75% of respondents agreed that more than 40% of their patients are achieving the treatment goals. However, HCPs consider maintaining healthy patient compliance as the most challenging part of the treatment (Figures 5B, 5C).

Response to control and treatment-based questions

**Figure 6:**
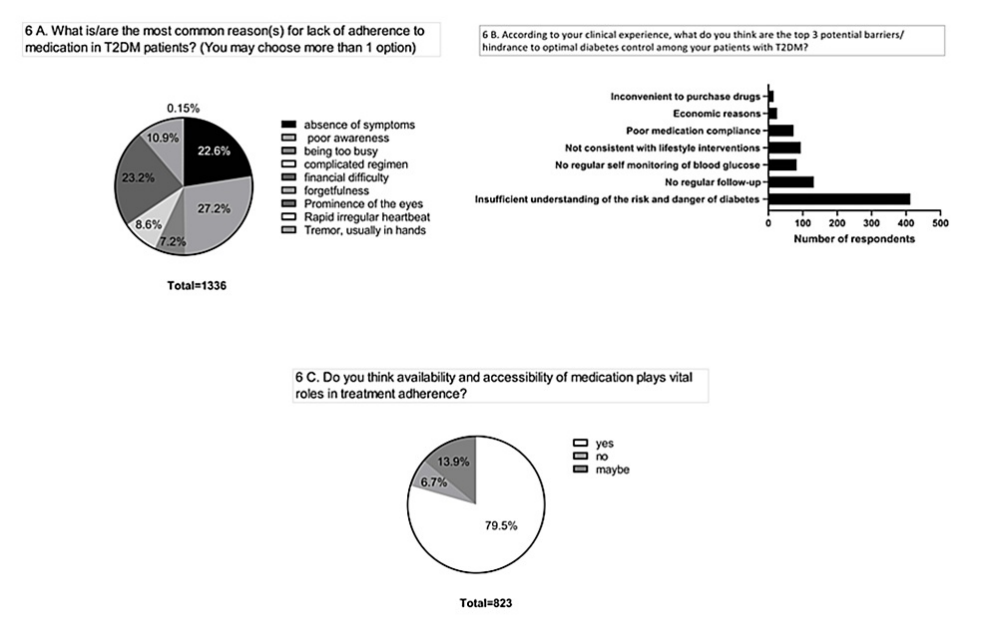
Treatment adherence. (A) Reasons for lack of adherence to medication. (B) Potential barriers to optimal diabetes control. (C) Availability and accessibility of medication. T2DM: type 2 diabetes mellitus.

Around three-fourths of the respondents opined that poor awareness, financial instability, and absence of symptoms were the major reasons for the lack of adherence to medication in patients with diabetes (Figure 6A). About 80% of the HCPs agree that insufficient understanding of the risks and dangers associated with diabetes is the most potential barrier to maintaining an optimal blood glucose level among patients. Lack of regular check-ups with doctors and unhealthy lifestyles among the patients pose a threat to achieving diabetes control (Figure 6B). About 80% of the HCPs feel that accessibility to medications would play an important role in adherence among the patients (Figure 6C).

**Figure 7:**
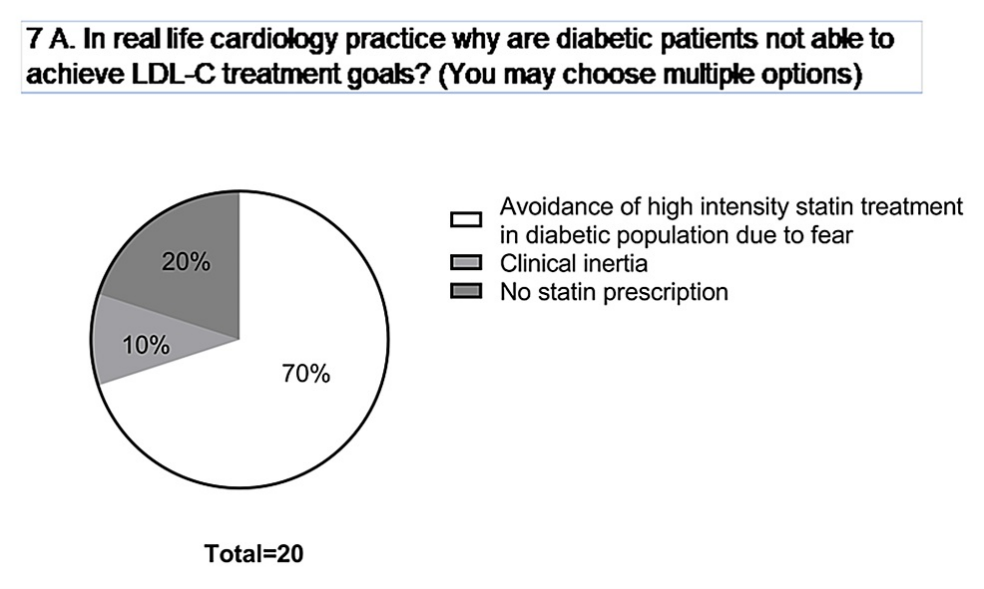
Reasons for not achieving LDL-C treatment by diabetic patients. LDL-C: low-density lipoprotein cholesterol.

Almost three-fourths of the patients (70%) tend to avoid high-intensity statin treatment due to fear (Figure 7). This was found to be one of the major reasons for diabetic patients not achieving low-density lipoprotein cholesterol (LDL-C) treatment. The major focus areas on which better awareness has to be created are mentioned in Table 2.

**Table 2:** Major focus areas for creating awareness among individuals. DM: diabetes mellitus; CVD: cardiovascular disease.

8. Focus areas on which better awareness has to be created	N (%)
Avoidance of high-intensity statin treatment in the diabetic population due to fear of drug interactions	4 (10.5)
Clinical inertia	3 (7.9)
No statin prescription	1 (2.6)
Choice of type of glucose-lowering medications and treatment targets	1 (2.6)
Cardiovascular risk assessment in patients with diabetes	4 (10.5)
Identifying patients with diabetes and those at risk of developing diabetes	1 (2.6)
Multifactorial treatment recommendations for patients with DM and CVD	3 (7.9)
Recommendations on lifestyle modification in diabetes	21 (55.3)

## Discussion

Our study aims to capture the experience and opinion of HCPs regarding the lack of awareness among Indian patients with diabetes. The broader aspiration of this study is to direct India toward being the "diabetes care capital" rather than being the diabetes capital of the world. The major theme of the study was to achieve a 90% goal of awareness and screening/detection, 90% of the diagnosed undergoing treatment, and 90% of the population with diabetes control. About 50% of the participating HCPs observed that only 40% of their patients with diabetes have sufficient awareness about the disease and its complications (Figure 1). Socioeconomic factors and inadequate accessibility to medicines were identified as the key barriers to this lack of awareness (Figure 6). Awareness regarding the complications associated with DM is necessary to encourage routine health check-ups among the population [6].

Appropriate awareness of the risk factors associated with DM is extremely important for implementing preventive measures. There is a great variation in the extent of awareness among different ethnic and social groups. A cross-sectional study [7] conducted in Bangladesh showed that 55% of the study individuals were unaware of hypertension as a risk factor for T2DM. In our study, only less than 40% of the patients were aware of the risk factors of diabetes. Less than 60% of their patients were aware of cardiovascular complications as a leading cause of death among T2DM patients. A study [8] among the African American ethnic population found that those with a family history of diabetes were more aware of the risk factors associated with diabetes and tried to incorporate certain changes in their lifestyle to prevent the incidence of the health condition. In our study, HCPs observe a significant difference in the prevailing extent of awareness between urban and rural populations. A community-based study [9] conducted in India observed that the urban population demonstrated relatively more awareness than those dwelling in rural areas. This underscores the need to organize awareness programs directed at improving the awareness level of the rural population regarding T2DM complications and management.

There is a unanimous opinion emphasizing the need for creating awareness among the Indian population irrespective of the medium. Almost all the HCPs suggest that it can be channeled via any mode, including direct counseling or training programs, social media or digital media-based programs, or posters. In a cross-sectional study [10], it was observed that the major source of spreading awareness of diabetes were the internet, social networks, and friends. Only less than 3% of the individuals depended on the medical staff for information regarding the management of diabetes. All of these reiterate the urgent need for HCPs to organize counseling or training programs in either offline or online mode.

To facilitate the process of helping individuals realize their glycemic status, monitoring of blood glucose levels shall be considered as a 5th vital for all patients during their hospital visits. A study in rural communities [11] found that screening for diabetes is very low among rural populations, and the monitoring of blood glucose levels among individuals with diabetes is less frequent.

Three-fourths of the respondents claim that the major reason for the under-diagnosis of diabetes could be due to a lack of awareness or financial constraints. A pan-India study [12] covering 50 districts and 25 states in India found that the average monthly health expenditure for the management of diabetes was ₹1357, which can be an economic burden for low to middle-income Indian families.

Although HCPs are the major player in spreading awareness of the need for regular follow-ups and regular monitoring of health status, they also recommend all the possible modalities for spreading awareness among the general population. Fear and avoidance of using statins impede attaining optimal levels of LDL-C among the patients (Figure 7). Since the majority of the population is unaware of the fatal consequences of cardiovascular disease (CVD) coexisting with diabetes, the lack of realization and the need for the spread of awareness extends to patients visiting cardiology and/or diabetes clinics (Figure 4).

Limitations

All the questions in the survey did not demand mandatory responses and involved HCPs practicing in different healthcare domains and practice settings. Hence, despite the inclusion of a large population of participants, we could get completed responses only from a subset of participants. The proportion of missing responses, therefore, varied from 1% to 99% for different questions. Not receiving a response has not been interpreted or considered for the analysis. The majority of the responses were based on the perception of the HCPs and were not based on a direct evaluation of the extent of knowledge among the patients. Some of the responses were not quantifiable, that elaborate inferences or interpretations were not possible.

## Conclusions

In a highly populous country like India, with disparities in socioeconomic status and healthcare access, there is a huge lag in the awareness of diabetes and its complications. Early recognition of an impending future illness burden is challenging. Routine screening for diabetes in the adult population over 30 years of age and in individuals with high risk for diabetes needs to be implemented.

## References

[1] American Diabetes Association (2011). Diagnosis and classification of diabetes mellitus. Diabetes Care.

[2] Davidson KW, Barry MJ, Mangione CM (2021). Screening for prediabetes and type 2 diabetes: US Preventive Services Task Force recommendation statement. JAMA.

[3] Yesudian CA, Grepstad M, Visintin E, Ferrario A (2014). The economic burden of diabetes in India: a review of the literature. Global Health.

[4] Dey S, Nambiar D, Lakshmi JK, Sheikh K, Reddy KS (2012). Health of the elderly in India: challenges of access and affordability. Aging in Asia: Findings From New and Emerging Data Initiatives.

[5] Caballero AE, Ceriello A, Misra A (2020). COVID-19 in people living with diabetes: an international consensus. J Diabetes Complications.

[6] Kaveeshwar SA, Cornwall J (2014). The current state of diabetes mellitus in India. Australas Med J.

[7] Mumu SJ, Saleh F, Ara F, Haque MR, Ali L (2014). Awareness regarding risk factors of type 2 diabetes among individuals attending a tertiary-care hospital in Bangladesh: a cross-sectional study. BMC Res Notes.

[8] Baptiste-Roberts K, Gary TL, Beckles GL, Gregg EW, Owens M, Porterfield D, Engelgau MM (2007). Family history of diabetes, awareness of risk factors, and health behaviors among African Americans. Am J Public Health.

[9] Sherlin M, Govindraj SC (2019). Awareness of diabetic complications among rural and urban diabetes population in Chennai. Int J Soc Rehabil.

[10] Shirmohammadi N, Soltanian AR, Borzouei S (2018). Public awareness of early and late complications of type 2 diabetes - application of latent profile analysis in determining questionnaire cut-off points. Osong Public Health Res Perspect.

[11] Fottrell E, Ahmed N, Shaha SK (2018). Diabetes knowledge and care practices among adults in rural Bangladesh: a cross-sectional survey. BMJ Glob Health.

[12] Nagarathna R, Madhava M, Patil SS, Singh A, Perumal K, Ningombam G, Nagendra AH (2020). Cost of management of diabetes mellitus: a pan India study. Ann Neurosci.

